# Pancreatic Lipase in Eutectogels as Emerging Materials: Exploring Their Properties and Potential Applications in Biosensing

**DOI:** 10.3390/bios15090615

**Published:** 2025-09-17

**Authors:** Raúl Martínez-Baquero, María José Martínez-Tomé, Javier Gómez, Rocío Esquembre, C. Reyes Mateo

**Affiliations:** Instituto de Investigación, Desarrollo e Innovación en Biotecnología Sanitaria de Elche (IDiBE), Universidad Miguel Hernández (UMH), 03202 Elche, Spain; raul.martinezb@umh.es (R.M.-B.); mj.martinez@umh.es (M.J.M.-T.); jgomez@umh.es (J.G.)

**Keywords:** eutectozyme, eutectogel, enzyme inhibition, anti-obesity drugs, orlistat, lipase, deep eutectic solvents

## Abstract

Eutectogels are advanced gel-based systems that integrate deep eutectic solvents (DES) into polymer networks. In this study, we report the first detailed characterization of an enzyme-containing eutectogel, representing a significant step toward advanced biosensing and biocatalytic applications. Specifically, we have incorporated pancreatic lipase, one of the main target enzymes in the treatment of obesity, in eutectogels via UV-induced radical polymerization of suitable precursors in appropriate DESs. Prior to immobilization, the enzyme was solubilized in selected DESs and its activity and conformational stability were evaluated using colorimetry and intrinsic fluorescence. Combinations of choline chloride/glycerol and tetramethylammonium chloride/glycerol were shown to be effective media for preserving and enhancing enzymatic function and conformational stability. The enzyme immersed in eutectogel exhibited high structural integrity and excellent thermal stability, maintaining its activity over several weeks. The ability of this new material to screen enzyme inhibitors was assessed using orlistat, a well-established anti-obesity agent. The results demonstrated clear detection of the drug’s inhibitory effect, even at nanomolar concentrations, highlighting the material’s potential as a screening platform for novel inhibitors with prospective anti-obesity activity. Furthermore, the device proved effective in quantifying drug presence, offering a promising and highly sensitive tool for pharmaceutical quality control applications.

## 1. Introduction

Deep eutectic solvents (DESs) are an emerging class of solvents that appeared in the early 2000s as a promising environmentally friendly alternative to conventional organic solvents and ionic liquids. They are commonly generated through the combination of hydrogen bond donors (HBDs) and acceptors (HBAs) at specific molar ratios, resulting in the formation of a stable and extensive hydrogen-bonding network with unique physicochemical properties. The cohesive network of interactions between these constituents allows such solvents to function as a unified entity, behaving as if it was a singular substance with a lower melting point than its pure individual components [1]. One of the most useful properties of DESs is their tunability, which allows the formulation of multiple combinations of HBAs and HBDs in different ratios, providing the opportunity to design rational solvents for specific purposes. In addition, most DESs are biodegradable, inexpensive, non-toxic, non-volatile and thermally stable and allow a wide range of solutes to be dissolved [2]. DESs can be confined in a 3D polymeric network, resulting in a versatile material, known as eutectogel (EG), which brings together the attributes of both, with properties that can be adjusted depending on the choice of components and their proportion. The polymeric network acts as structural support for the DES, providing mechanical stability to the material, as well as elasticity and stretchability. Such properties make EGs attractive for a variety of applications, making them a promising tool in fields such as medicine, food, energy and the environment [3,4,5,6,7].

There is also a growing interest in using DESs as a greener and cost-effective alternative in biocatalysis and biosensing applications. Numerous studies have demonstrated that enzymes, such as lipases, laccases and proteases, can not only retain but even exhibit enhanced activity and stability in certain specific DESs, used as solvents or co-solvents, compared to the control [8,9,10]. Concerning the mechanisms behind enzyme stabilization and activation in DESs, most research suggests that enzyme performance is primarily influenced by the overall DES complex rather than through the individual components or their synergistic action. Nevertheless, the composition of DESs plays a vital role in enzymatic activity and stability, as these properties have been shown to vary across different DES types, depending on the nature of HBAs and HBDs and their molar ratio [1,11].

Given the benefits that DESs offer enzymes and the widely recognized fact that the immobilization of proteins on suitable supports represents an attractive way to improve their stability and facilitate their reuse, it is to be expected that the incorporation of proteins in EGs constitutes a very advantageous alternative that will enable the creation of functional materials with unique properties, especially in the field of biocatalysis and biosensing. Nevertheless, very few studies have explored or implemented this approach to date [12,13]. Recently, our group has developed and characterized fluorescent nanocomposite EGs to which alkaline phosphatase has been incorporated. This work is one of the first demonstrations of the maintenance of the activity of an enzyme in a eutectogel and led to the development of a prototype of fluorescent biosensor for detecting hydrolase activity [14]. Recent studies have also reported eutectogels for biosensing applications [7]; however, these devices cannot be regarded as biosensors, because they do not incorporate a biological recognition element.

Lipases are part of the family of hydrolases that act on acylglycerols to liberate fatty acids and glycerol. In addition to their hydrolytic activity, they are able to catalyze interesterification, esterification, aminolysis and alcoholysis reactions. These versatile enzymes, especially microbial ones, have been extensively studied in DESs, showing good compatibility, often maintaining or even increasing their catalytic efficiency. Immobilization techniques have also been widely employed to enhance their operational stability, reusability and overall performance, with a primary focus on developing more sustainable and effective processes across a range of industrial fields, including biodiesel production, biocatalysis and organic synthesis [15,16,17]. Nevertheless, despite the potential advantages for various applications, there are currently no published studies of lipases being immobilized within EGs, an approach that would enable both dissolution in DESs and immobilization.

Another application of lipases is focused on the search for new drugs. Specifically, pancreatic lipase (PL) has been used on numerous occasions, both immobilized and in solution, as an important target for screening compounds that inhibit its activity, which could act as potential anti-obesity drugs. Since this enzyme, secreted by the pancreas, acts in the human digestive system by catalyzing the hydrolysis of triglycerides and other carboxylic esters into monoacylglycerols and free fatty acids, effective inhibitors may help modulate fat absorption in the intestine [18,19]. Pancreatic lipase obtained from porcine pancreas (porcine pancreatic lipase, PPL) is commonly used as a model enzyme for this purpose, due to its commercial availability and stability and its higher amino acid sequence homology (86%) with human PL [19,20]. It is a small globular protein composed of a single chain of 449 amino acids and a molecular weight of ~50 kD, with a catalytic triad constituted by Ser153, Asp177, and His264, defining the active site of the enzyme. This active site is highly restricted by a hydrophobic lid domain which controls both enzyme activation and substrate specificity. The lid changes from closed (inactive) to open (active) conformation when the enzyme is exposed to the hydrophobic media or hydrophobic/hydrophilic interfaces. This conformational change allows substrates to enter the active site and be hydrolyzed [21,22,23].

As compared to microbial lipases, PPL is one of the least studied lipases in DESs. Recently, Rashid et al. reported that this enzyme was significantly activated in the presence of a ternary DES medium composed of choline chloride (ChCl), glycerol (Gly) and water and hypothesized that ternary DES binds to the enzyme at a spot other than the active site, inducing conformational changes, leading to lipase activation when hydrated with water molecules [24]. In contrast, Elgharbawy et al. reported a lower relative activity compared to the control when the enzyme was dissolved in different types of menthol-based hydrophobic DESs [25].

Taking all the above into consideration, this study aims to investigate the feasibility of immobilizing PPL within an eutectogel and to comprehensively evaluate its performance. This includes not only assessing its catalytic activity, but also examining the properties of the new material, its stability, storage capacity, and overall suitability for potential biotechnological applications. Specifically, we will focus on exploring whether this material can be used to detect ligands with deleterious effect on enzyme activity, which could potentially become anti-obesity drugs. To this end, the enzyme was previously dissolved in different DES compositions, and its behavior (conformation, stability and activity) was characterized in order to select the DESs with the optimal performance for the enzymatic assays. The results were partly compared with those of lysozyme, a model enzyme widely studied in these solvents [11]. This comparison is particularly relevant as both are hydrolases that rely on water as a substrate. In particular, three DESs were synthesized by the thermal method using quaternary ammonium salts as HBAs, namely, ChCl and tetramethylammonium chloride (TMAC), and Gly or urea as HBDs.

EGs were prepared through UV-induced in situ radical polymerization of suitable precursors in appropriate DES [14]. Specifically, the monomer 2-hydroxyethyl methacrylate (HEMA) was dissolved in the selected DES, in absence and presence of PPL, along with the crosslinker ethylene glycol dimethacrylate (EGDMA) and the photoinitiator. Once prepared, the EGs were analyzed in terms of rheological properties, stability and swelling behaviour and the enzyme activity inside the eutectogel was also evaluated. Finally, the resultant new material was tested as a platform for the detection of enzyme inhibitors, using as model tetrahydrolipstatin (orlistat), a commercial anti-obesity drug.

## 2. Materials and Methods

### 2.1. Reagents

The following materials were procured from Sigma-Aldrich (Merck Life Science, Madrid, Spain): 2-hydroxyethyl methacrylate (HEMA) and ethylene glycol dimethacrylate (EGDMA) (both stabilized with hydroquinone monomethyl ether for synthesis), 2,4,6-trimethylbenzoylphenylphosphinate lithium salt (LiTPO), choline chloride (ChCl), tetramethylammonium chloride (TMAC), glycerol (Gly), urea, porcine pancreatic lipase (PPL) (EC 3.1.1.3; Type II, lyophilized, Mw = 48,000 Da), p-nitrophenyl acetate (pNPAc), p-nitrophenyl palmitate (pNPP), and orlistat.

TRIS buffer (100 mM, pH 8.2) was prepared by dissolving TRIS acid in Milli-Q water, with pH adjustment using sodium hydroxide. Milli-Q water (twice-distilled and deionized) was obtained using a Milli-Q IQ 7000 ultra pure water system (Merck, Millipore^®^, Madrid, Spain). All solvents used were of spectroscopic or analytical grade (Merck, Uvasol^®^, Madrid, Spain).

The enzyme aqueous solutions were prepared immediately before use, by suspending crude porcine pancreatic lipase powder in TRIS buffer to give a concentration of 84 mg/mL (4368 units/mL). The solution was then centrifuged at 6000  rpm for 10 min in order to eliminate undissolved material and the clear supernatant was recovered.

### 2.2. Preparation of Deep Eutectic Solvents (DES)

DESs were prepared by combining the following components in a 1:2 molar ratio: ChCl and urea (ChCl-urea), ChCl and Gly (ChCl-Gly) and TMAC and Gly (TMAC-Gly). Each mixture was stirred at 80 °C until a clear, homogeneous liquid was obtained [11,26].

### 2.3. Preparation of Eutectogels (EGs) and Eutectozymes

**Scheme 1:**
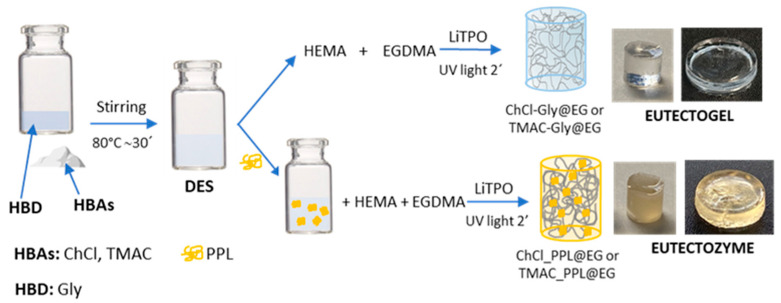
Preparation of Eutectogels and Eutectozymes.

Two different types of EGs were prepared via radical polymerization, following the protocol described by Alacid et al. with slight modifications [14]. In the synthesis of the first EG, denoted as ChCl-Gly@EG, the monomer HEMA was dissolved in ChCl-Gly in presence of the crosslinked agent EGDMA and the photoinitiator LiTPO, while in the second eutectogel, named TMAC-Gly@EG, the DES used for the preparation was TMAC-Gly with the rest of the components remaining the same. The eutectogels with the immobilized enzyme were referred to as eutectozymes, following the terminology by Kumar et al. [12] and, depending on the DES used in their preparation, they have been designed as ChCl_PPL@EG or TMAC_PPL@EG (Scheme 1).

In a typical preparation of EGs, 600 µL of the precursor HEMA, 600 µL of the corresponding DES and 5 µL EGDMA were combined in a vial and thoroughly stirred. Under dark conditions, 2 mg of LiTPO were added to the mixture and stirred until completely dissolved. Polymerization was then initiated by exposing the solution to UV light at 365 nm for 2 min. For the preparation of eutectozymes, 0.1 g of PPL were incorporated during the mixing step together with HEMA, EDGMA, LiTPO and the corresponding DES, resulting in a protein concentration of 1.75 mM in the eutectogel.

It should be noted that the final geometry and size of the EGs, both in the presence and absence of PPL, were tailored according to the specific experimental requirements. Thus, for protein activity assays, the entire volume of the mixture (1205 μL) was irradiated in a 20 mm diameter vial, obtaining disk-shaped materials of ~20 mm diameter by ~5 mm thickness, which were cut into six identical portions. In contrast, for rheological characterization, 400 μL of the initial mixture were dispensed into tubular molds of 8 mm of base and after irradiating the sample, cylindrical eutectogels of ~8 mm diameter by ~8 mm height were obtained.

### 2.4. Fluorescence Measurements

Fluorescence spectra measurements were performed by placing the samples in 10 × 10 mm quartz cuvettes using a PTI-QuantaMaster spectrofluorometer (PTI, Birmingham, NJ, USA) equipped with a Peltier cell. Emission spectra were recorded with an excitation wavelength of 290 nm and an emission range between 300 and 440 nm.

Thermal stability of PPL was determined by recording emission spectra as a function of temperature and plotting the fluorescence intensity ratio at different wavelengths (335 nm and 375 nm) over the course of the thermal denaturation curve. Heating rates of 2.5, 5 and 10 °C·min^−1^ were applied, with stabilization times of 30 s at each temperature. The thermal denaturation temperature T_m_ of ALP was calculated by plotting the first derivative of the thermal denaturation curve. The final T_m_ value corresponds to the mean of three samples [27].

### 2.5. Activity Assay in Solution

PPL activity assays were performed in triplicate using colorimetry to monitor the time-course production of p-nitrophenol (pNP), obtained by the PPL-catalyzed hydrolysis of either pNPAc or pNPP substrates. Measurements were conducted at both room temperature and at 37 °C using a UV-2700 spectrophotometer (Shimadzu, Tokyo, Japan). Enzyme solutions in buffer, in pure DES or in mixtures of DES and buffer, at a concentration of 175 μM and 2 mL as final volume, were placed in quartz cuvettes with a 10 mm path length, and absorbance values were recorded over time at 405 nm, where pNP exhibits a strong absorption peak. The kinetics of the catalyzed reaction was monitored plotting the % of pNP formed (obtained using molar extinction coefficient, ε^405^ = 1.8 × 10^4^ M^−1^⋅cm^−1^) versus time [28].

For both pNPAc and pNPP, stock solutions were prepared at a concentration of 3 mM in isopropanol. In the case of pNPAc, an initial 1:10 dilution was made in buffer (resulting in 300 µM), and subsequently, 200 µL of this intermediate solution was added to the final reaction volume of 2 mL. Therefore, the final concentration of pNPAc was 30 µM, and the isopropanol content in the final mixture was 1% *v*/*v*. In contrast, pNPP is a more hydrophobic substrate and requires a higher proportion of isopropanol in the final 2 mL reaction volume to ensure its complete dissolution. To determine the optimal conditions, activity assays were performed using various final isopropanol concentrations. These included 0% isopropanol, where pNPP was directly dissolved in buffer (although it appeared insoluble), 1% isopropanol to match the conditions used with pNPAc, and progressively higher concentrations, up to 50%, selecting 20% *v*/*v* as the optimal concentration for activity assays (Figure S6). Therefore, all activity experiments using pNPP as substrate (both for free and immobilized enzyme) were carried out with this final concentration of isopropanol.

### 2.6. Activity Assay in Eutectozymes

To evaluate the activity of the enzyme encapsulated in the eutectogel, a disk-shaped eutectozyme (ChCl_PPL@EG or TMAC_PPL@EG) was prepared in triplicate and sectioned into six equal pieces, as previously described, each with an approximate volume of 200 µL. Prior to measuring the activity, each of these portions was introduced in a quartz cuvette, together with 1600 µL of buffer solution, and shaken for 15 min in an orbital shaker. Afterwards, the mixture was incubated for 5 min at 37 °C inside the spectrophotometer, before initiating the reaction with the addition of 200 µL of substrate. Note that the initial concentration of 1.75 mM of the enzyme within the eutectogel was reduced to an effective concentration of 175 µM, considering the total 2 mL volume (200 µL of eutectogel plus 1800 µL of solution). The time-course of the hydrolysis reaction was monitored by measuring the formation of pNP in the solution throughout the time.

### 2.7. Inhibition Assays in Eutectozymes

For the inhibition assays, the commercial anti-obesity drug orlistat was chosen as the model compound due to its well-established competitive inhibitor of pancreatic lipase [29]. Stock solutions of this inhibitor were prepared at a concentration of 40 µM in isopropanol. The assay conditions were analogous to those described in the previous section, with the exception that the 1600 µL of solution incubated with the eutectozyme contained the inhibitor in solution. The inhibition assays were performed in triplicate at final orlistat concentrations ranging from 0 to 700 nM.

### 2.8. Swelling Measurements

Swelling measurements of ChCl-Gly@EG and TMAC-Gly@EG were performed in triplicate both with and without the inclusion of PPL as is described in Alacid et al. [14]. Freshly prepared eutectogels were immersed in an excess of Milli-Q water, approximately 20 mL, at room temperature. The initial weight of each sample ($W_{0}$) was recorded, and subsequent weights ($W_{t}$) were measured at regular intervals as the eutectogels absorbed water (every 10 min the first hour, every 20 min the second hour, every hour for six hours and at 24 h after the immersion). The degree of swelling (*SW*) was calculated using the following equation:

(1) $$SW=\frac{W_{t}-W_{0}}{W_{0}}$$

### 2.9. Rheological Measurements

Duplicates of ChCl-Gly@EG and TMAC-Gly@EG, both with and without PPL, were prepared and rheological measurements were conducted using dynamic mechanical analysis (DMA) performed on a DMA 1 Instrument (Mettler-Toledo). The instrument performs DMA calculations based on the force and displacement measurements to derive the viscoelastic material parameters [30]. The tests were carried out at 25 °C using compression platens. The EGs were cylindrical in geometry with diameters of ~8 mm and a height of 8 mm. Strain sweeps between 0.1% strain and 3.3% strain were performed at 1 Hz. Concretely, the samples are forced to undergo an oscillatory deformation in compression of 5, 10, 20, 50, 75, 100 and 200 μm for 2 min at each deformation and their storage modulus (E‘) and loss modulus (E”) obtained from the force required to produce that deformation and the geometry of the sample are recorded. Frequency sweeps from 0.1 Hz to 50 Hz were performed at strain level of 0.33%, in the linear viscoelastic region.

### 2.10. Thermogravimetric Analysis (TGA)

The thermal stability of the EGs was investigated in the 25–500 °C temperature range by dynamic thermogravimetric analysis (TGA) and differential thermogravimetric analysis (DTG) using a TGA/DSC 3+ (Mettler Toledo) apparatus operating under N_2_ atmosphere at a heating rate of 10 °C min^−1^, as is described in Rezić et al. [31]. Mass change was recorded as a function of temperature. The weight samples range was 17 ± 3 mg.

## 3. Results and Discussion

### 3.1. Conformational Stability of PPL in DES

The primary structure of PPL contains seven tryptophan residues that are responsible for its intrinsic fluorescence, five of which are located in its N-terminal domain. In particular, Trp30 is partially exposed to the solvent, and lies close to the active site of the enzyme and is known to be the major contributor to the total emission of the protein [32,33]. Given the high sensitivity of tryptophan fluorescence to the polarity of the local microenvironment, we used the intrinsic fluorescence of the enzyme as a tool to monitor the conformational alterations induced by the incorporation of the protein into the selected DESs. In order to determine the most suitable protein concentration for this study, solutions in TRIS buffer with increasing concentrations of PPL were prepared, and the emission spectra were recorded (Figure S1a). The position and shape of this spectrum coincide with those previously described in the literature for this enzyme, confirming that the protein is in its folded conformation, in the closed (inactive) state [32]. As expected, the fluorescence intensity increased with concentration. However, when plotting the maximum emission intensity versus concentration, a loss of linearity was observed, especially above 10 μM of PPL, so we selected 5 μM as the optimal concentration for the following fluorescence studies.

**Figure 1:**
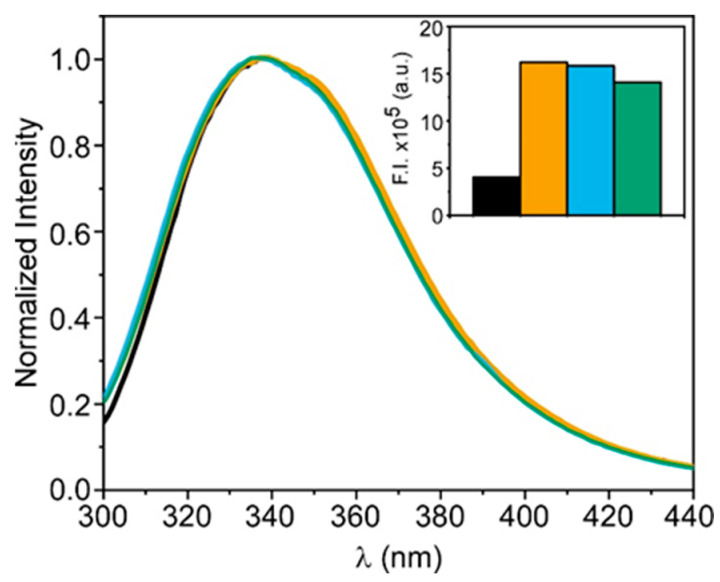
Normalized fluorescence emission spectra recorded at room temperature (λ_ex_ = 290 nm) of PPL in solution (black), ChCl-Gly (blue), TMAC-Gly (green) and ChCl-urea (orange). Inset: Bar chart depicting the maximum fluorescence intensity of each spectrum prior to normalization.

Fluorescence emission spectra of the enzyme were obtained in the DES composed of ChCl-Gly, TMAC-Gly and ChCl-urea and compared to those recorded in TRIS buffer (Figure 1). To decrease the viscosity of the samples, aqueous dilutions of the DES with a 75 *v*/*v* % concentration were prepared. For these highly concentrated DES mixtures, the native hydrogen bonding and the nanostructure of the DES are practically maintained, but viscosity is greatly reduced, which facilitates the dissolution of the enzyme [34]. For all the samples the shape of the fluorescence spectrum was quite similar, indicating that the native structure of the protein is maintained in the DES. The small spectral blue shift (around 2 nm) observed in the ChCl-Gly, TMAC-Gly DES could reflect the location of tryptophanyl side chains in an environment of lower polarity as previously reported for lysozyme [11]. It should be noted that the fluorescence intensity was not similar in all cases (inset in Figure 1), being approximately four times higher in the three DES systems studied, probably due to the inherent high viscosity associated with these solvents as well as the decrease in polarity [32,35].

The intrinsic fluorescence of PPL was also used to examine conformational alterations of the enzyme upon thermal unfolding. In the native folded state, most of the tryptophan residues are found within the protein core, whereas in a partially folded or unfolded state, they are exposed to the solvent resulting in a red shift in the emission spectra. For this reason, monitoring the fluorescence spectra with temperature and plotting the ratio of intensities at 335 and 375 nm (I_335_/I_375_) is a good strategy to explore the thermal stability of PPL, in order to determine whether its incorporation into the DES can affect this stability. This study was first carried out in buffer, between 10 and 86 °C, at different scanning speeds (2.5, 5 and 10 °C/min). The obtained curves, displayed in Figure S1b, were very similar for the three selected rates and the results suggest that the protein is very sensitive against temperature, as was previously reported using circular dichroism [36]. As can be seen in Figure S1b, the I_335_/I_375_ ratio was practically constant up to 45 °C. Between 45 and 50 °C it diminished very slightly and above this temperature it decreased abruptly, with a midpoint temperature (T_m_) of 58 ± 2 °C, which suggests that the protein unfolds through a two-state process, as has been reported for other hydrolases and for the PPL against chemical denaturants [11,36]. When the sample was cooled from 86 °C to room temperature, the value of the I_335_/I_375_ ratio was not recovered, indicating that the protein was unable to refold to its native conformation after cooling, in agreement with the results obtained by circular dichroism [36].

**Figure 2:**
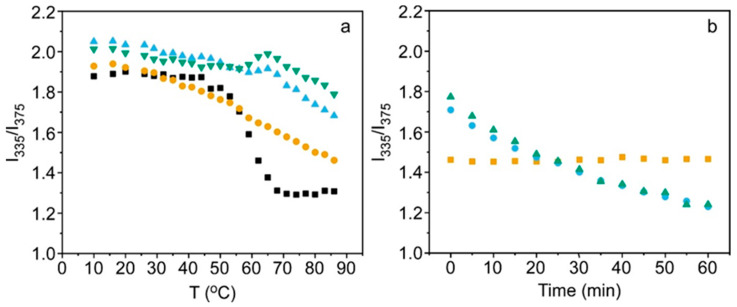
Thermal stability of PPL in buffer (black), ChCl-Gly (blue), TMAC-Gly (green) and ChCl-urea (orange), monitored by changes in I_335_/I_375_ fluorescence intensity ratio as a function of temperature (**a**) and its evolution during 1 h at 86 °C, after temperature scan (**b**).

Thermal denaturation experiments of the protein performed in three DESs showed results very different from those observed in buffer (Figure 2a). In the case of ChCl-urea, the I_335_/I_375_ ratio decreased from 20 to 86 °C with very little cooperativity. This nearly linear decrease suggests that PPL is denatured through an energetically close ensemble of folding intermediates rather than the two-state process observed in buffer solution. A similar result was reported for lysozyme dissolved in the same eutectic solvent [11]. In contrast to this result, we found the effect of temperature on the unfolding of the protein in ChCl-Gly and TMAC-Gly DES to be much smaller. The intensity ratio barely decreased as the temperature increased up to 60 °C, and even experienced a slight rise between 60 and 65 °C, while from 65 to 86 °C the decrease was more pronounced. This behaviour suggests that ChCl-Gly and TMAC-Gly contribute to maintaining the native conformation of the protein, likely by forming a protective layer around it that fosters the folded conformation. Besides this thermodynamic stabilization, the DES may also contribute to kinetic stabilization by slowing down the rate of unfolding. This could be due to reduced conformational flexibility needed for unfolding and/or because the high viscosity of these solvents significantly slows down structural rearrangements. As a result, the unfolding process becomes much slower than the natural folding dynamics of the protein. To investigate whether this kinetic stabilization occurs when PPL is dissolved in the DES, additional experiments were performed. In particular, once the temperature scan was completed for the three DESs, at a scanning speed of 2.5 °C/min, the protein was maintained at 86 °C and the denaturation kinetics of the enzyme was monitored for one hour (Figure 2b). The results show that in the presence of ChCl-urea, the protein reached its maximum unfolded state immediately after the thermal scan. In contrast, in the presence of ChCl-Gly and TMAC-Gly, the enzyme required more than 60 min to complete the denaturation process. This result is consistent with those obtained for lysozyme, where urea-based DES showed greater protein structure destabilization upon thermal treatment compared to the Gly-based DES, which is indicative of the destabilizing character of this eutectic mixture [11]. Results also evidence that the unfolding kinetics in ChCl-Gly and TMAC-Gly are significantly slower than the applied heating rate, indicating that these solvents probably exert a strong kinetic stabilization effect on PPL.

To confirm the above hypothesis, PPL was dissolved in neat DESs of ChCl-Gly and TMAC-Gly, in order to increase viscosity, and the emission spectra and temperature scans were recorded (Figure S2a–c). To facilitate the dissolution of the protein, the sample was heated, maintaining the temperature below its denaturation temperature. The results show that, on the one hand, the emission spectra, especially those of the protein in pure TMAC-Gly, are slightly blue-shifted compared to those obtained for aqueous dilutions of the DES with a 75% *v*/*v* concentration. This result is likely attributed to a decrease in the polarity of the medium caused by water loss, and it is more pronounced in the DES containing TMAC, which has lower polarity than ChCl due to the absence of a hydroxyl group in its molecular structure.

On the other hand, the temperature scans, although exhibiting greater dispersion, showed that even at 86 °C, the protein maintains its folded structure, evidencing the kinetic stabilization previously reported.

### 3.2. Enzyme Activity of PPL in DES

After studying the conformational stability of PPL in DESs, we investigated whether the protein remained catalytically active when dissolved in ChCl-Gly and TMAC-Gly, while ChCl-urea was discarded for this study in view of its effect on the PPL thermal stability. The enzyme activity was measured as described in Materials and Methods, using p-nitrophenyl acetate (pNPAc) as substrate and monitoring the kinetics of formation of p-nitrophenol (pNP) which absorbs at 405 nm. To carry out the experiment, we selected a protein concentration in buffer capable of transforming in pNP all the added substrate (30 μM) in a few minutes. Increasing concentrations were tested from 0 to 175 μM finding that at the latter protein concentration, pNPAc was completely hydrolyzed in 4 min, a time that seemed adequate to carry out our study (Figure S3.)

**Figure 3:**
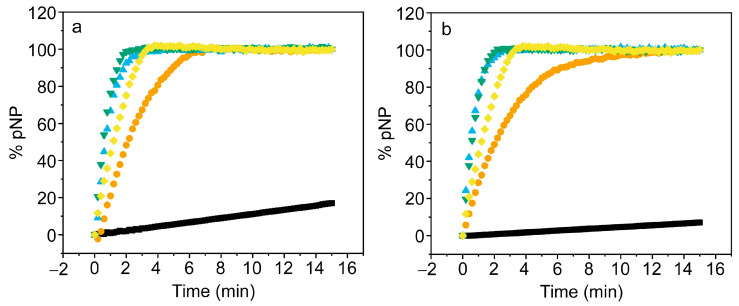
PPL activity, expressed as percentage of pNP formation after addition of pNPAc (30 μM), measured in neat DES (black) and in DES:buffer mixtures of (**a**) ChCl-Gly and (**b**) TMAC-Gly, with decreasing DES content (*v*/*v*): 75% (orange), 50% (blue), 25% (green), and 0% DES (yellow).

With this PPL concentration, we analyzed the enzyme activity in the neat DES as well as in aqueous eutectic mixtures with DES content changing from 75 to 0% *v*/*v*. As can be seen in Figure 3, PPL hydrolytic kinetics in pure DESs were extremely slowed down as compared to the activity in buffer. Similar results were reported for lysozyme in neat ChCl:Gly [11]. This low catalytic activity may be ascribed to a combination of factors. Firstly, the elevated viscosity of the reaction in the presence of DESs may impose significant diffusional limitations, thereby reducing the rate of substrate accessibility and hindering product release. Secondly, the high viscosity could constrain the conformational dynamics of the enzyme, which is essential for the progression of its catalytic cycle. Additionally, the limited availability of water in the system may be deleterious for the hydrolytic mechanism, further contributing to the reduced enzymatic efficiency. The fact that the activity lost in the presence of DESs was recovered upon dilution supports these hypotheses. In fact, the activity not only increased significantly upon dilution but also, at a certain proportion of DESs in the medium (25–50% *v*/*v*), it was even higher than that observed in buffer alone. A similar effect has been described in other studies for lipases other than pancreatic lipase when dissolved in water-DES mixtures. The hypothesis formulated in those works and which could be applicable to PPL is that the DES components bind to the enzyme at a site other than its active site, inducing conformational changes that shift the equilibrium towards the open conformation of the lipase, favouring the catalysis reaction [24].

Regardless of the mechanism responsible for this behavior, the results obtained so far indicate that the dissolution of PPL in neat DESs composed of TMAC-Gly or ChCl-Gly thermally stabilizes the enzyme and maintains it in a structurally stable conformation, even if not optimally active, that protects it from denaturation. The enzyme can be efficiently reactivated when diluted with buffer to a DES concentration of 25–50% (*v*/*v*), its activity being even higher than that observed in buffer. Thus, the findings suggest that the DES would be a suitable medium to preserve the enzyme, although for its complete functionality, it must be diluted with an appropriate amount of water.

### 3.3. Characterization of PPL in Eutectogels

**Figure 4:**
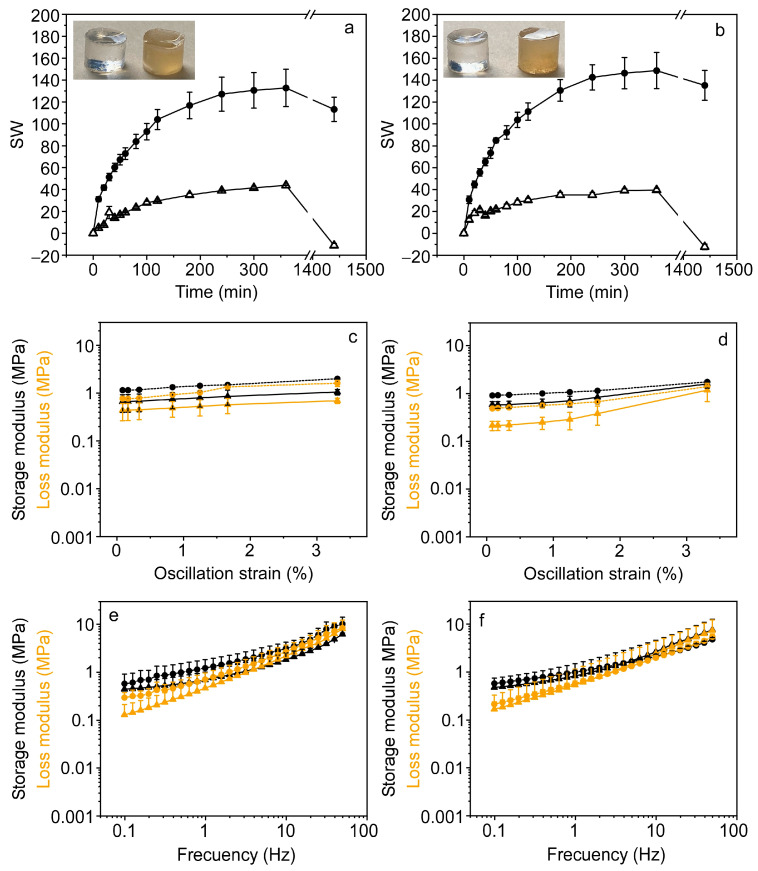
Swelling behavior and rheological properties of ChCl-Gly@EG (**a**,**c**,**e**) and TMAC-Gly@EG (**b**,**d**,**f**) in the absence (triangle) and in the presence (circle) of PPL. Insets: Digital image of ChCl-Gly@EG (**a**) and TMAC-Gly@EG (**b**) without (left) and with (right) PPL obtained before swelling. Representative plots of strain (**c**,**d**) and frequency (**e**,**f**) sweep measurements at 25 °C for EG (triangle) and PPL@EG (circle). Error bars represent the standard deviation of two replicates.

After characterizing the behavior of PPL in glycerol-based DES and demonstrating their benefits, the next step was to obtain EGs containing the enzyme. Such materials, known as eutectozymes, have hardly been investigated. Before proceeding to immobilization, EGs were obtained in the absence of the enzyme and their properties were characterized. EGs were synthesized by light-induced radical polymerization dissolving HEMA monomer and the crosslinking agent EGDMA in the pure DES of TMAC-Gly and ChCl-Gly. The obtained materials, TMAC-Gly@EG and ChCl-Gly@EG, were transparent and stable, as shown in the insets of Figure 4a,b and Scheme 1.

To get insight into their mechanical behavior, oscillatory rheology measurements were performed, as is described in Materials and Methods. The strain sweep plots, performed at 1 Hz (Figure S4a and Figure 4c,d), show that for both types of EGs, the storage modulus E’, which reflects the elasticity of the material, is higher than the loss modulus E” (the viscous component) over the whole tested range of oscillation strain, especially below 2%. This result suggests that both TMAC-Gly@EG and ChCl-Gly@EG behave mainly as elastic solids capable of regaining their initial form once the stress is eliminated, without disrupting or changing their internal structure. At low oscillation strain, the storage moduli were very similar for both eutectogels, while the ratio of the storage modulus to the loss modulus, the so-called damping factor (tan δ) was 0.6 and 0.4 for TMAC-Gly@EG and ChCl-Gly@EG, respectively. Although still less than 1, these values are higher than those observed for eutectogels of similar formulation, likely due to the lower amount of crosslinking agent used in their preparation [14]. The frequency sweep plots, displayed in Figure S4b and Figure 4e,f show that the storage modulus values were much less frequency dependent than those of the loss modulus, up to ~3 Hz, evidencing that the EGs show a good tolerance to the applied strain rate of deformation below this frequency. However, above 3 Hz there is a crossover point, so the elastic and viscous behaviors are comparable, suggesting that the EGs are in a transition zone where neither solid nor liquid behavior is clearly dominant [37,38].

The thermal stability of EGs was evaluated by TGA. As shown in Figure S4c, the TGA and corresponding derivative thermogravimetric (DTG) curves display four distinct stages of weight loss, each evidenced by a peak in the DTG profiles. Both eutectogels demonstrated high thermal stability, retaining approximately 90–95% of their initial weight at 100 °C. This first weight loss is attributed to the evaporation of physically adsorbed water and is commonly observed in systems containing deep eutectic solvents (DES), due to their inherently hygroscopic nature [39]. The second stage of mass loss, observed between 200 and 300 °C, corresponds to the evaporation of glycerol—the hydrogen bond donor (HBD) in both DES formulations—as well as to the degradation or volatilization of unreacted monomers (HEMA and EGDMA), and low-molecular-weight fragments, possibly arising from incomplete polymerization or thermal degradation of the crosslinked network [40]. The third stage, centered around 300 °C, is assigned to the thermal decomposition of the ionic components of the DESs. These include ChCl and TMAC in ChCl-Gly@EG and TMAC-Gly@EG, respectively. Both salts are known to undergo decomposition at elevated temperatures, contributing significantly to mass loss during this stage [40,41]. Finally, the fourth and most prominent decomposition stage occurs above 400 °C and is attributed to the breakdown of the covalent bonds in the crosslinked polymeric network formed by HEMA and EGDMA. This step represents the thermal decomposition of the organic matrix and results in near-complete mass loss [14].

It is known that polymer gels, immersed in a suitable solvent, are able to swell to reach a volume several times their initial one. Since EGs are very novel materials, their degree of swelling has not been studied in detail as compared, for example, to that of hydrogels. The swelling behavior of EGs in water provides insight into the affinity of these materials towards aqueous media and thus determines to what extent they can be used for applications in aqueous solutions. With this in mind, we determine the swelling degree, SW, of the synthesized EGs in water, as is described in Materials and Methods (Equation (1)). For this experiment, a piece of eutectogel of ~1 g was immersed in 50 mL of Milli-Q water, and its mass was measured as a function of time. The results plotted in Figure 4a,b show that both, TMAC-Gly@EG and ChCl-Gly@EGs, absorb water molecules increasing their mass in a similar way: the first few minutes very rapidly and then increasingly more slowly, reaching a plateau after 3–4 h. When the material was weighed after 24 h incubation in aqueous solution, a decrease with respect to the initial weight was observed in both EGs. This behavior was very different from that observed in many hydrogels, where the gain in mass is enormous and stabilizes after several hours [27,42]. The lower degree of swelling and weight loss seems to be a particular feature of some eutectogels, since a very similar behavior was recently observed in HEMA eutectogels prepared with ChCl-ethylene glycol DES. In that work it was suggested that after a certain immersion time, DES starts to exchange for water molecules, leading to mass reduction [14].

For the incorporation of PPL into EGs to obtain eutectozymes, the enzyme was mixed with the precursor, crosslinker agent and photoinitiator using DES ChCl-Gly and TMAC-Gly as solvents, as is described in Materials and Methods. Irradiation of the mixture gave rise to materials named, respectively, ChCl_PPL@EG and TMAC_PPL@EG, which retained a similar appearance to the starting EGs in terms of shape, but with higher opacity and a yellowish coloration (Inset in Figure 4a,b and Scheme 1). This appearance is due to the high concentration of enzyme, which was estimated based on the activity results obtained in DES, under the assumption that the eutectogel would retain the enzyme in a stable and immobilized form capable of performing its catalytic function once the substrate is added. Therefore, it was calculated that for a final volume of 2 mL including buffer, the enzyme concentration in the eutectogel should be 1.72 mM.

The effects of the incorporation of PPL on the rheological behavior of EGs are shown in Figure 4c–f. Results suggest that PPL is integrated into the polymer network of both EGs, slightly reinforcing it and augmenting internal friction without fundamentally altering the overall viscoelastic behavior of the composite material. Specifically, ChCl_PPL@EG and TMAC_PPL@EG, especially the former, exhibited increased stiffness and elasticity, indicated by the higher storage modulus value, and greater energy dissipation reflected in the more elevated loss modulus value. However, while both elastic and viscous were amplified, the fundamental manner in which EGs reacted to varying stresses and frequencies remained rather consistent with the original material.

The swelling behavior of ChCl_PPL@EG and TMAC_PPL@EG was explored and compared with that observed for EGs in absence of proteins. The results show a relatively similar pattern of behavior, but the maximum degree of swelling was much higher for the eutectozymes, especially for TMAC_PPL@EG (Figure 4a,b). This behaviour could be caused by the high concentration of PPL. This enzyme, like many proteins, has hydrophilic regions on its surface that could interact favorably with water, facilitating its entry into the EG and promoting swelling, creating a more hydration-favorable environment within the material.

### 3.4. Enzyme Activity of PPL in Eutectogels

**Figure 5:**
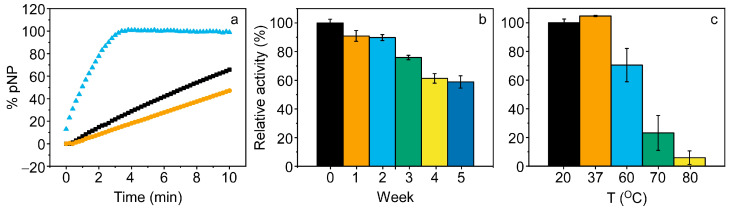
PPL activity expressed as percentage of pNP formation after addition of pNPAc (30 μM), measured in buffer (blue), TMAC_PL@EG (black) and ChCl_PL@EG (orange) (**a**). Effect of storage time on the relative activity of TMAC_PL@EG (**b**). Thermal stability of TMAC_PPL@EG evaluated by measuring residual activity after 5 min incubation at various temperatures (**c**). Error bars represent the standard deviation of three replicates.

Although we can be certain that the enzyme is entrapped in the eutectogel, given that polymerization occurs in its presence, the immobilization protocol may promote protein unfolding, so that the enzyme would no longer show catalytic activity. In this case, and unlike what was conducted in DES, it was not possible to assess the conformational state of the enzyme via fluorescence measurements due to interference from the eutectogel matrix. Therefore, we proceeded directly to evaluate its enzymatic activity. To carry out this assay a disk-shaped eutectozyme (ChCl_PPL@EG or TMAC_PPL@EG) was prepared and sectioned into six equal pieces as described in Materials and Methods. One of these portions was placed in a cuvette containing buffer solution and stirred to allow the EG to swell and create optimal conditions for substrate diffusion. Then, an aliquot containing the substrate pNPAc (final concentration 30 μM) was added to the cuvette and the increase in absorbance of the solution was measured at 405 nm. As can be seen in Figure 5a, while for PPL in buffer all the added substrate is hydrolyzed in ~4 min, for ChCl_PPL@EG or TMAC_PPL@EG the hydrolysis reaction was not completed even after 10 min of incubation. The decrease in the rate of the catalyzed reaction was expected and might be attributed to diffusion restrictions imposed by the polymeric network, which limit the accessibility of pNPAc to the active site of the enzyme, although some loss in the intrinsic activity of the enzyme cannot be ruled out. Similar behaviour has been observed for other enzymes, such as alkaline phosphatase, immobilized in both hydrogels and sol–gel matrices [27,43]. This result confirms that the enzyme after being immobilized in eutectogels is still functional and suggests that the enzymatic response is better in TMAC_PPL@EG than in ChCl_PPL@EG, since the hydrolysis efficiency is higher in the former.

Due to the interesting results obtained with TMAC_PPL@EG we explored the stability of this material with storage time. For this purpose, a new disk-shaped EG was prepared and cut into 6 identical portions. The enzymatic activity of the first portion was evaluated on the same day of preparation by determining the slope of the linear portion of the progress curve, which represents the formation of pNP over time. The remaining portions were stored under refrigeration and analyzed at weekly intervals. As shown in Figure 5b, the enzyme activity was very similar during the first 15 days and after 35 days the material still maintained more than 60% activity.

The effect of temperature on TMAC_PPL@EG was also explored by measuring the enzyme activity after incubation of the material for 5 min at 20 °C, 37 °C, 60 °C, 70 °C and 80 °C (Figure 5c). In contrast to what was observed in buffer (Figure S5), the enzyme remained active at 60 °C, well above its denaturation temperature. Even at 70 °C it was able to maintain some activity, behaviour likely caused by both the kinetic and thermodynamical stabilization exerted by DES on the native conformation of the enzyme.

### 3.5. TMAC_PPL@EG as Platform for Biosensing Applications

The above results indicate that PPL can be incorporated into eutectogels while probably retaining its functional structure and allowing the enzyme to be efficiently reactivated upon buffer addition, with TMAC_PPL@EG offering the best performance. As mentioned previously in the introduction, one of the possible applications of PPL immobilization is focused on the search for new inhibitors that can act as potential anti-obesity drugs. With this in mind, we evaluated to what extent the developed eutectogel could be used for this purpose. To conduct this study, we selected p-nityrophenylpalmiate (pNPP) instead of pNPAc as the enzyme substrate of PPL and kinetics were recorded at 37 °C. pNPAc, employed in the previous experiments, is a substrate commonly used for the initial characterization of the catalytic activity of this enzyme, as well as for comparative studies. However, the hydrolysis of pNPAc, due to its short chain (C2), may not fully reflect the hydrolysis of long-chain triglycerides, which are the natural substrates of PPL. In contrast pNPP, with its palmitate chain (C16) provides a more physiologically relevant and accurate representation of PPL activity, making it the preferred substrate for inhibition studies.

The use of pNPP as a model hydrophobic substrate required the inclusion of a water-miscible organic cosolvent to improve substrate availability in the aqueous phase, as is described in Material and Methods. In this context, varying concentrations of isopropanol were assessed to facilitate micelle formation and thereby enhance the interaction between the substrate and the PPL active site. The results showed that in aqueous solution, in the absence of cosolvent no activity of the enzyme was detected though the insolubility of the substrate may be responsible for the observed behavior. However, as reaction media contained increasing concentrations of isopropanol, a dramatic growth in enzyme activity was detected, reaching a maximum at 20% (*v*/*v*) alcohol content. (Figure S6). Higher concentrations of isopropanol again decreased the activity, probably by inhibiting micelle formation, solubilizing the substrate, or even denaturing the enzyme. Thus, a 20% isopropanol concentration appears to be optimal, not only by enhancing substrate solubility and promoting substrate formation, but also by facilitating lipase interfacial activation more effectively than in pure aqueous conditions, likely favouring the open, active conformation necessary for efficient catalysis.

After selecting the substrate conditions, the ability of the TMAC_PPL@EG platform to screen for enzyme inhibitors was explored using orlistat, a well-established anti-obesity drug [44]. This molecule is a pancreatic lipase inhibitor that acts by covalently binding to the serine residue of the lipase active site, preventing hydrolysis of substrates. According to the assay protocol outlined in the Section 2, three disk-shaped eutectogels were produced to allow for triplicate testing and each was divided into six equal segments. The enzymatic activity of all portions was evaluated on the same day of preparation by the progress curve, which represents the formation of pNP over time.

**Figure 6:**
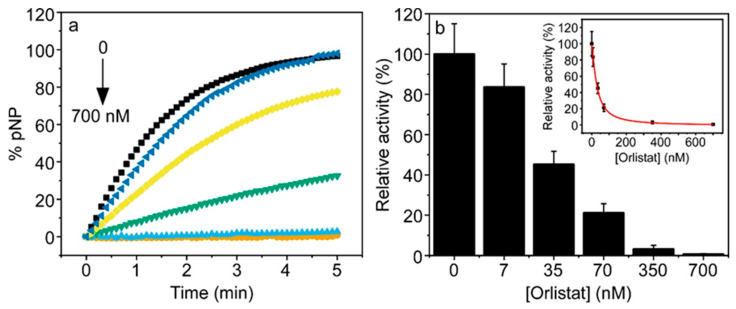
Effect of increasing concentrations of orlistat: 0 (black), 7 (navy), 35 (yellow), 70 (green) 350 (blue) and 700 (orange) nM, on the activity of TMAC_PPL@EG expressed as percentage of pNP formation after addition of pNPP (60 μM) (**a**). Relative activities of TMAC_PPL@EG derived from the linear slopes of the activity curves (**b**) and inset. Error bars represent the standard deviation of three replicates.

For each disk, one portion was incubated without orlistat and used as control, while the other five were incubated with increasing concentrations of the inhibitor, up to 700 nM. As shown in Figure 6a, as the concentration of orlistat increases, the rate of pNPP hydrolysis decreases, until the reaction is completely inhibited above 350 nM. This result clearly shows that the device is capable of detecting the inhibitory effect of the drug and therefore supports its potential application as a screening platform for the identification of novel inhibitors with prospective anti-obesity activity.

Figure 6b highlights the above conclusion. The plot displays the relative PPL activities, averaged from triplicate inhibition assays, derived from the linear slope of the activity curves shown in Figure 6a. Based on the remaining enzymatic activity, a dose–response prediction can be generated by fitting the experimental values to a logistic regression model, as illustrated in the inset of Figure 6b. This approach allows us to determine a specific inhibition value using our system, which opens the door to future comparisons between newly identified potential inhibitory compounds.

Furthermore, the data obtained in Figure 6b (inset) could be used to quantify the concentration of orlistat in a given sample, since the graph represents the calibration curve of the compound. This information is of interest, among other things, for the good manufacturing practice of pharmaceutical products, to ensure correct dosing in tablet formulations, as well as to assess their shelf life. Our platform could be a very sensitive, simpler, more cost-effective and faster alternative to the current methods used for the determination and stability testing of this compound, which are currently performed by HPLC [44].

## 4. Conclusions

In this work, eutectogels were successfully synthesized via UV-induced radical polymerization of 2-hydroxyethyl methacrylate (HEMA) and ethylene glycol dimethacrylate (EGDMA) in DES composed of either TMAC-Gly or ChCl-Gly. Porcine pancreatic lipase (PPL) was immobilized within these matrices, representing one of the first reported cases of enzyme incorporation in such materials. Prior to immobilization, PPL was characterized in both DES, where it was found that DES thermally stabilize PPL and maintain its conformational integrity, despite limited catalytic activity in the neat solvent. Enzymatic activity was efficiently restored, and even improved, upon dilution with buffer to DES concentrations of 25–50% (*v*/*v*), supporting the use of DES as effective preservation media when adequate hydration is ensured. The eutectogels displayed elastic solid behavior, with the incorporation of PPL slightly increasing internal friction without altering the overall viscoelastic response. The swelling of the material in water was significantly enhanced in enzyme-loaded eutectogels, likely due to favorable interactions between water and hydrophilic regions on the protein surface.

Eutectogels composed of TMAC-Gly had slightly favorable enzymatic properties compared to those made of ChCl-Gly, so these stable and well temperature-resistant materials were selected to fabricate a biosensing platform to detect enzyme inhibitors, which could potentially be used as anti-obesity drugs. The resulting device, tested using orlistat as a model drug, demonstrated clear detection of the inhibitory effect, even at nanomolar concentrations. In addition, the device also proved effective in quantifying the presence of the drug, offering a promising and highly sensitive tool for pharmaceutical quality control applications.

In summary, this study presents the first comprehensive characterization of an enzyme-containing eutectogel, demonstrating its potential as a robust and versatile platform for biosensor applications. Beyond these findings, the tunable nature of eutectogel matrices offers interesting opportunities for further optimization of enzyme performance and the development of advanced biofunctional devices. These prospects highlight the broad applicability and promising potential of eutectozymes in next-generation biotechnologies.

## Data Availability

The original contributions presented in this study are included in the article/Supplementary Material. Further inquiries can be directed to the corresponding author(s).

## Supplementary Materials

The following supporting information can be downloaded at: https://www.mdpi.com/article/10.3390/bios15090615/s1, Figure S1: Fluorescence emission spectra and thermal stability of PPL in buffer; Figure S2: Fluorescence emission spectra and thermal stability of PPL in DES; Figure S3: PPL activity in buffer as a function of protein concentration; Figure S4: Plots of strain and frequency sweep measurements and TGA for ChCl-Gly@EG and TMAC-Gly@EG; Figure S5: Residual activity of PPL in buffer and TMAC-Gly@EG, after temperature incubation; Figure S6: Effect of the isopropanol content on the activity of PPL.

## Abbreviations

The following abbreviations are used in this manuscript:

DES	Deep eutectic solvent
HBD	Hydrogen bond donor
HBA	Hydrogen bond aceptor
EG	Eutectogel
PL	Pancreatic lipase
PPL	Porcine pancreatic lipase
ChCl	Choline chloride
Gly	Glycerol
TMAC	Tetramethylammonium chloride
HEMA	2-Hydroxyethyl methacrylate
EGDMA	Ethylene glycol dimethacrylate
LiTPO	2,4,6-Trimethylbenzoylphenylphosphinate lithium salt
pNPAc	p-Nitrophenyl acetate
pNPP	p-Nitrophenyl palmitate
pNP	p-Nitrophenol
*W*_0_	Initial weight
*W_t_*	Subsequent weight
*SW*	Degree of swelling
DMA	Dynamic mechanical analysis
E‘	Storage modulus
E’’	Loss modulus
TGA	Thermogravimetric analysis
DTG	Differential thermogravimetric analysis
T_m_	Midpoint temperature
Tan δ	Damping factor
