# Supplementary material for: Pancreatic Lipase in Eutectogels as Emerging Materials: Exploring Their Properties and Potential Applications in Biosensing
Source: Biosensors (Basel). 2025 Sep 17;15(9):615. doi: 10.3390/bios15090615 (PMC12467884; doi:10.3390/bios15090615)
Supplement: Supplementary file 1 [file biosensors-15-00615-s001.zip › biosensors-3843238-supplementary.pdf]

biosensors

MDPI

Supplementary materials

Pancreatic Lipase in Eutectogels as Emerging Materials: Exploring Their Properties and Potential Applications in Biosensing

Raúl Martínez-Baquero [1], María José Martínez-Tomé [1], Javier Gómez [1], Rocío Esquembre [1,*] and C. Reyes Mateo [1,*]

[1] Instituto de Investigación, Desarrollo e Innovación en Biotecnología Sanitaria de Elche (IDiBE), Universidad Miguel Hernández (UMH), 03202 Elche, Spain; raul.martinezb@umh.es (R.M.B.); mj.martinez@umh.es (M.J.M.-T.); jgomez@umh.es (J.G.).

* Correspondence: resquembre@umh.es (R.E.); rmateo@umh.es (C.R.M.)

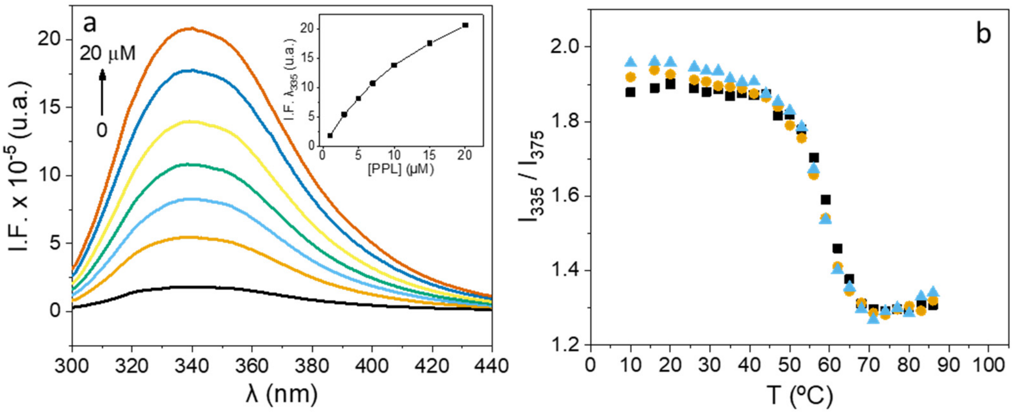

Figure S1. Fluorescence emission spectra (λ_x = 290 nm) of PPL in TRIS buffer as a function of increasing concentrations (0–20 µM). Inset: Fluorescence intensity recorded at 335 nm, at increasing concentrations of PPL (**a**). Effect of temperature on the I_{335}/I_{375} intensity ratio of PPL in buffer measured at 2.5 °C/min (blue), 5 °C/min (orange) and 10 °C/min (black) heating rates (**b**).

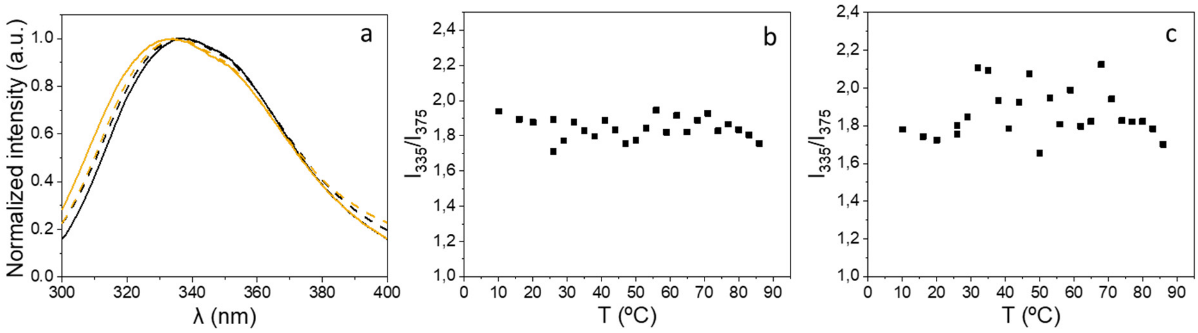

Figure S2. Normalized fluorescence emission spectra of PPL dissolved in ChCl-Gly 75% (dashed line black) and 100% (dashed line orange), and TMAC-Gly 75% (solid line black) and 100% (solid line Orange) (**a**). Temperature scans of PPL dissolved in pure ChCl-Gly 100% (**b**) and TMAC-Gly 100% (**c**).

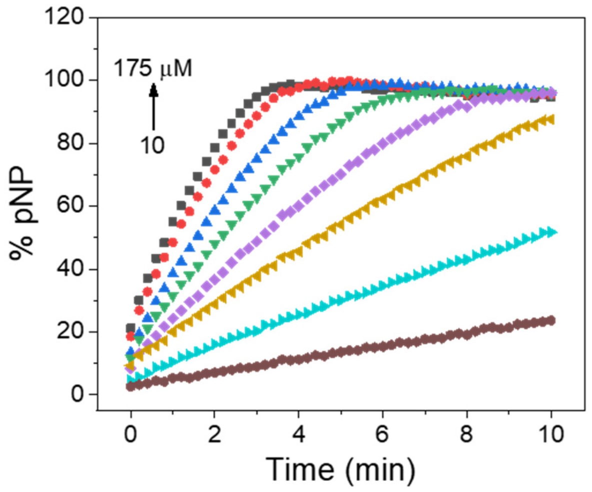

Figure S3. PPL activity in TRIS buffer expressed as percentage of pNP formation after addition of pNPAc (30 µM), as increasing concentrations of protein: 10 µM (brown), 25 µM (light blue), 50 µM (yellow), 75 µM (purple), 100 µM (green), 125 µM (dark blue), 150 µM (red) and 175 µM (black).

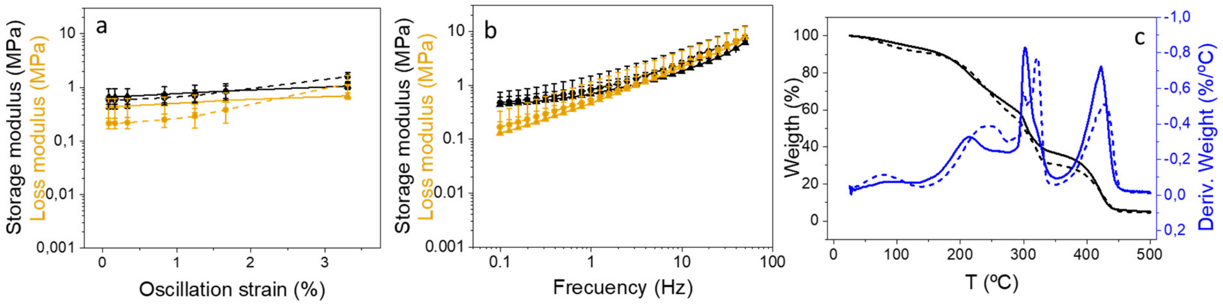

Figure S4. Representative plots of strain (**a**) and frequency (**b**) sweep measurements at 25 °C for ChCl-Gly@EG (triangle) and TMAC-Gly@EG (circle). TGA (black) and DTG (blue) curves of ChCl-Gly@EG (solid line) and TMAC-Gly@EG (short dashed line) (**c**). Error bars represent the standard deviation of two replicates.

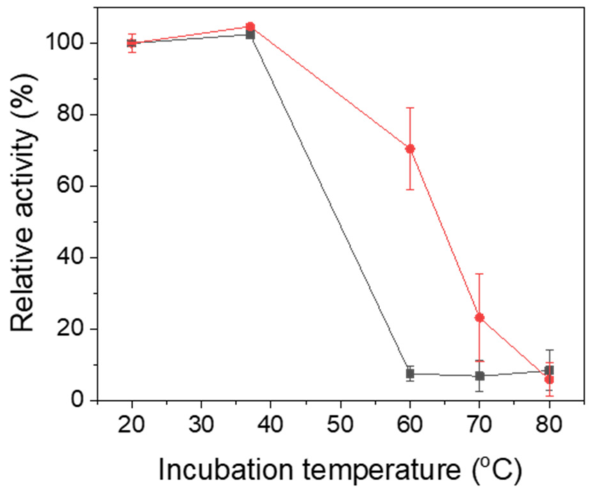

Figure S5. Thermal stability of PPL in buffer (black) and TMAC_PPL@EG (red), evaluated by measuring residual activity after 5-min incubation at various temperatures.

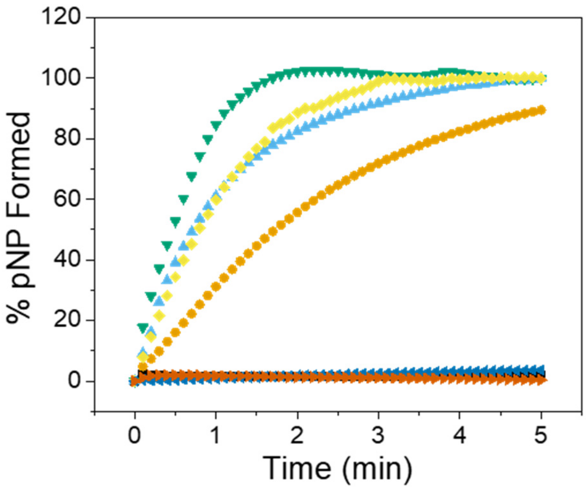

Figure S6. PPL activity expressed as percentage of pNP formation after addition of pNPP (30 μM), dissolved in isopropanol:buffer mixtures with increasing isopropanol content (v/v): 0% (black), 1% (orange), 10% (blue), 20% (green), 30% (yellow), 40% (dark blue) and 50% (red).
